# Impact of COVID-19 on Patients with a Preferred Language Other than English in the Emergency Department

**DOI:** 10.5811/westjem.18610

**Published:** 2025-07-09

**Authors:** Molly Thiessen, Emily Hopkins, Jennifer Whitfield, Kristine Rodrigues, David Richards, Leah Warner, Jason Haukoos

**Affiliations:** * Denver Health Medical Center, Department of Emergency Medicine, Denver, Colorado; † University of Colorado School of Medicine, Department of Emergency Medicine, Aurora, Colorado; ‡ University of Colorado School of Medicine, Department of Pediatrics, Aurora, Colorado; § Colorado School of Public Health, Department of Epidemiology, Aurora, Colorado

## Abstract

**Background:**

The COVID-19 pandemic had a disproportionate impact on minority communities, including patients who identify as having a preferred language other than English (PLOE). Our primary goal in this study was to evaluate the effect of the COVID-19 pandemic on patients with a PLOE in the emergency department (ED), and the use of interpreter services. Secondary outcomes evaluated were measures of patient care, including length of stay, number of studies performed, and unplanned return visits to the ED.

**Methods:**

We performed an interrupted time series study of prospectively collected electronic health record (EHR) adult ED and language services data from an urban, safety-net hospital.

**Results:**

The total number of patients presenting to the ED went down in the early peak of the pandemic; however, the percentage of patients with a PLOE went up compared with previous years (19% vs 16%) and, despite making up only 19% of total patients, comprised 44% of total COVID-19 positive patients. In-person interpreter use decreased (prevalence ratio 0.49, 95% confidence Interval [CI] 0.43–0.56) while telephonic and video interpretation increased (prevalence ratio 3.97, 95% CI 3.56–4.43). Baseline testing was unchanged. All groups experienced a decrease in median LOS in 2020, but this was only found to be significant for patients who speak a language other than English or Spanish (P<0.001). None of the patient groups experienced a significant increase in unscheduled returns in 2020.

**Conclusion:**

Our data confirms that COVID-19 disproportionately affected patients with a PLOE, with patients with a PLOE 2.9 times more likely to test positive for COVID-19 than their English-speaking counterparts. Efforts should be made to mitigate this effect via language-concordant care, professional interpreters, and culturally appropriate interaction and information dissemination, not only as it relates to planning for public health crises, but in the day-to-day function of the healthcare system at large. Continued research into the factors driving these inequities and ways to mitigate them is warranted.

## INTRODUCTION

The COVID-19 pandemic emerged worldwide as a major health concern. Its impact on minority communities highlighted healthcare disparities at many levels.^1^ In particular, its impact on patients who have a preferred language other than English (PLOE) has been significant.^2^–^7^ The United States is home to a large number of people who speak a language other than English at home, including 21.5 million people who identify as having a PLOE.^8^ Patients who identify their preferred language as something other than English are more likely to contract COVID-19, have lower testing rates, and are more likely to be hospitalized with COVID-19.^9^–^13^

While a study in a pediatric emergency department (ED) demonstrated increased interpreter utilization during the pandemic,^13^ to our knowledge there are no published studies looking at the effects of COVID-19 on PLOE patients, the use of interpreter services, and the effects of these factors on patient care in an adult ED setting. Multiple lay sources have reported decreased availability of in-person interpreters, difficulty in coordinating phone and video interpreters in a short time frame, and limitations in the use of all forms of interpreters in the setting of ubiquitous mask and other personal protective equipment (PPE) use.^2^–^6^ The current body of evidence clearly demonstrates that use of professional interpreters and/or language-concordant care result in fewer communication errors, improved patient comprehension, improved clinical outcomes, and equalization of healthcare and clinical services utilization.^14^,^15^ Unfortunately, the setting of COVID-19 complicated the use of professional interpreters, as described in the lay press.

Our primary goal in this study was to evaluate the association between COVID-19 and adult ED patients with a PLOE. Secondary goals were to evaluate the utilization of interpreter services and the associated measures of patient care, including length of stay (LOS), number of diagnostic studies performed, and unplanned return visits to the ED, during the first peak of the pandemic.

## METHODS

### Study Design and Setting

We performed an interrupted time series study of prospectively collected electronic health record (EHR) (Epic Systems, Inc, Verona, WI) ED and language services data from Denver Health Medical Center in Denver, Colorado. Denver Health Medical Center is an integrated health system and anchor institution for the County of Denver that includes an acute care hospital, Denver Health Medical Center, with a high-volume adult and pediatric ED, a Level I trauma center, and multiple community-based clinics. Denver Health Medical Center serves large numbers of patients from minority, underinsured, homeless, and immigrant communities. This study was approved by our institutional review board as being exempt from informed consent and is reported in accordance with STROBE guidelines for electronic data extraction.^16^

### Population

We included all adult (≥18 years of age) patients who presented to the ED from April 1–April 30 from 2017–2020. The month of April was chosen as it included the initial peak of the COVID-19 pandemic in the US in 2020 and allowed comparison to prior non-pandemic years. At the time of this analysis, subsequent months had significantly lower COVID-19 volumes; for this reason we analyzed this initial peak.

**Population Health Research Capsule**

What do we already know about this issue?

*The COVID-19 pandemic had a global health impact, disproportionately affecting minority communities*.

What was the research question?

*What is the association between COVID-19 and emergency department (ED) patients who prefer a language other than English, as well as patterns of interpreter use?*

What was the major finding of the study?

*Patients who preferred a language other than English were more likely to have COVID-19 than their English-speaking counterparts, OR 2.9 (95% CI 2.1–4.1)*.

How does this improve population health?

*We found an association of COVID-19 with language itself, not just race or ethnicity, which should inform efforts toward language equity in the ED and from a public health standpoint*.

### Data Collection

Variables extracted from the EHR included demographics (age, gender, race, ethnicity, address, and primary preferred language); payer status; chief complaint; top five discharge diagnosis codes (per the *International Classification of Diseases 10^th^Revision [ICD-10]*); laboratory studies performed; radiology studies performed; LOS; and return visit to the ED within 48 hours.

Interpreter services data were obtained from the hospital’s Department of Language Services. Language services at Denver Health Medical Center are comprised of in-house interpreters who can interpret in person or via telephone, as well as contracted services from an external vendor that provides interpretation via telephone or video. We queried the in-house log for in-person interpreters for the specified time frame; the contracting entity that provided external telephone and video interpretation also provided logs for these services over the specified time period.

### Outcomes

The primary outcome was diagnosis of COVID-19, defined as the presence of the ICD-10 code for COVID-19 (U07.1). We summarized interpreter service data into two service categories: 1) the use of language line services when ED staff would call into a vendor providing interpreter services over the phone or via video; or 2) in-person interpreter services when dedicated hospital language services staff were consulted to provide face-to-face interpretation between medical staff and the patient. Emergency department LOS was defined as the number of minutes between time of ED arrival and time of ED departure. We stratified ED visit characteristics (LOS; number of lab or radiology studies; return visits within 48 hours by the primary preferred language of the patient (categorized as English, Spanish and other) and reported across the four years of the study period.

### Data Management and Statistical Analyses

We extracted visit-level data using structured query language (SQL Server Management Studio, Microsoft Corporation, Redmond, WA) and transferred into an Excel spreadsheet (Microsoft Corporation, Redmond, WA). We performed data management and analyses using SAS Enterprise Guide v 8.3 (SAS Institute, Inc, Cary, NC). Descriptive statistics for continuous data are reported as means with standard deviations for normally distributed data and medians with interquartile ranges (IQR) for non-normally distributed data, and categorical data are reported as counts, proportions, or percentages with 95% confidence intervals (CI). Bivariate statistical tests (eg, Wilcoxon rank-sum test, chi-square) were used to compare variables and absolute differences with 95% CIs reported between groups. For the primary analysis, we used a multivariable logistic regression model to assess the association between PLOE and COVID-19 diagnoses and included the following covariates as potential confounders: age; sex; race; ethnicity; and Area Deprivation Index (ADI), which was used to adjust for socioeconomic context. The ADI is a validated, composite measure of US area-based deprivation based on 17 variables on poverty, education, housing, and employment.

We matched the geographic locations of patient-reported address for each visit to census tracts (CT). The ADI was calculated for each populated tract in Denver County from the 2015–2019 American Community Survey five5-year estimates (US Census.gov). We assigned each Denver CT an ADI by weighting the 17 variables by the factor score coefficients. The index was standardized with a mean of 100 and a SD of 20 and divided into quintiles as the ADI, consistent with other studies that included ADI, and because it has not been validated as a continuous predictor.^17^ Higher ADI index values were indicative of higher deprivation. Patients who were homeless did not have an address to geocode and were assigned an ADI value of “most deprived.” We assessed effect modification between Hispanic ethnicity and PLOE by including an interaction term into the logistic model. The primary explanatory variable was a PLOE, defined as a patient-reported preferred language as something other than English. The primary unit of analysis was the visits. Given that this was an observational study with no explicit testable hypothesis, no a priori sample size calculation was performed.

The data provided for interpreter services included information about each encounter, and there may have been multiple interpreter-service encounters for the same patient. The provided data did not include a patient identifier to allow for linkage of each interpreter-service encounter with a specific patient visit. Thus, results are presented as total number of interpreter-service contacts (language line or in person) per year (numerator) and number of ED visits (denominator). We calculated prevalence ratios of interpreter-service contacts and ED visits with April 2017 serving as the reference.

## RESULTS

The total number of ED visits for the month of April ranged from 3,557 (in 2020) to 4,952 (in 2019). Patient demographics are shown in Table 1. The median percentage of patient visits with a PLOE in April 2017–2019 was 16% (2,275/14,646), whereas in April 2020 these patients represented 19% of all visits (683/3,557). The proportion of visits in April 2020 with a COVID-19 diagnosis was 7% (261/3,557). Demographics of patients based on their COVID-19 diagnosis are shown in Table 2. Of the 261 patient visits with COVID-19 diagnosis, 114 (44%) had a PLOE. Of patients who identified their primary language as English, 5% (95% CI 4–6%) (147/2,875) had a final diagnosis of COVID-19. Among those patients who identified as PLOE, 18% (95% CI 15–21%) (102/559) of patients who preferred Spanish and 10% (95% CI 5–10%) (12/121) of patients who preferred other languages had a final diagnosis of COVID-19.

**Figure 1:**
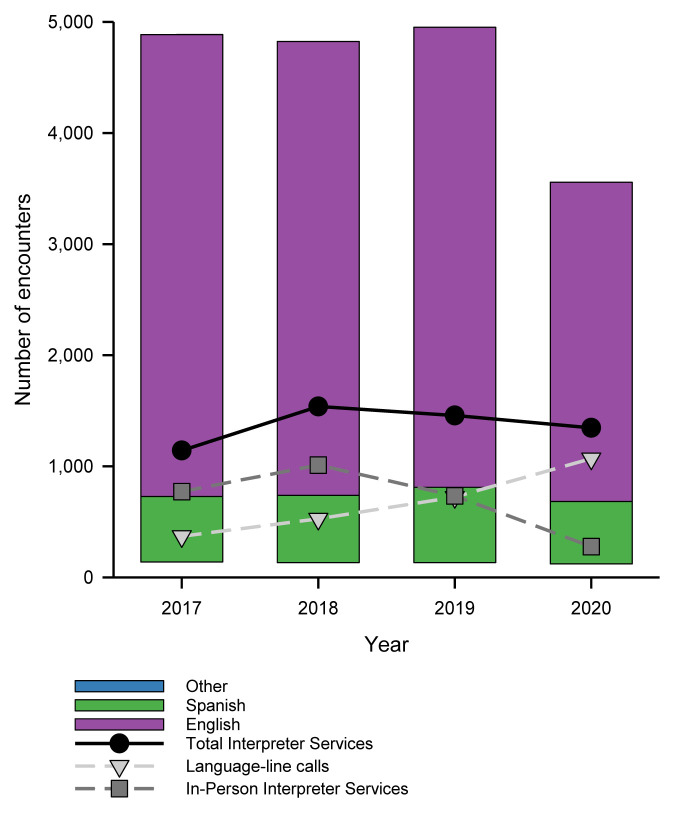
Number of emergency department visits and interpreter service encounters during the month of April between 2017–2020. The number of interpreter service encounters includes situations in which there may have been more than one interpreter encounter per patient.

A significant association was identified between patients with a PLOE and a COVID-19 diagnosis (Table 3). Interaction between Hispanic ethnicity and a PLOE was not significant (*P* = 0.3) and, thus, was not included in the final model. The number of contacts with interpreter services as they relate to total ED visits are displayed in Figure 1.

**Figure 2:**
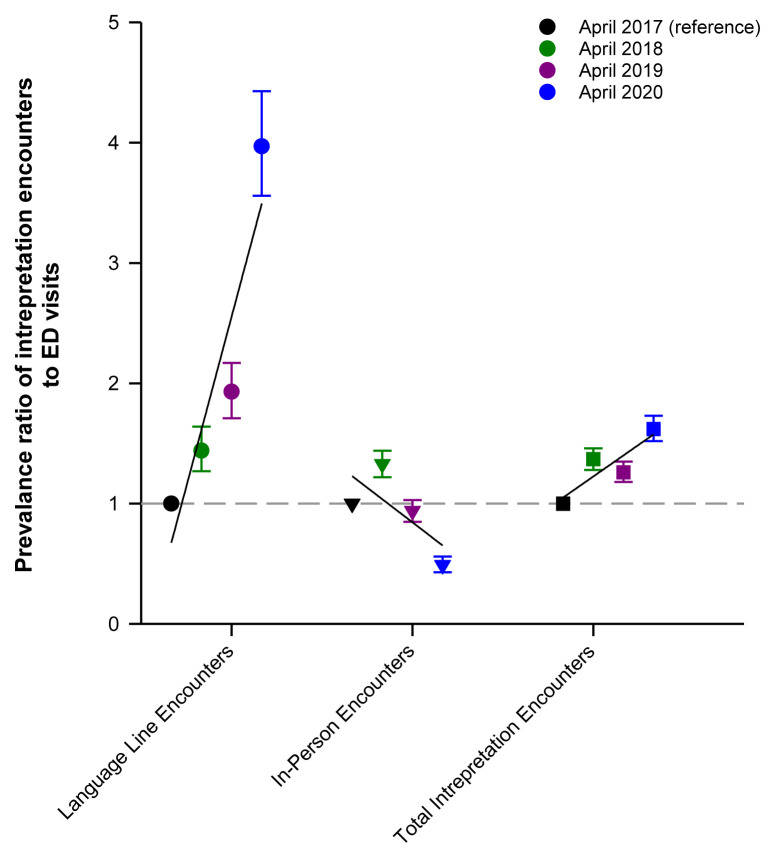
Prevalence ratio of interpreter encounters to ED visits.

The prevalence ratio of total interpretation encounters per patient was significantly higher in April 2020 compared to April 2017 (Figure 2 and Table 4). The prevalence ratio of remote (video and telephonic) interpreter encounters went up in April 2020, while the prevalence ratio of in-person interpreter encounters went down (Figure 2).

The other outcomes measured, including median number of lab studies, median number of radiology studies, median ED LOS, and unplanned return visits within 48 hours are shown in Table 5, with *P*-values in Table 6. The median number of radiology studies performed per ED visit was 1, and there was no significant difference among the groups. The median number of lab studies ordered per ED visit was four for Spanish-speaking patients, and three for patients who spoke English or other languages. This difference was only significant for Spanish-speaking patients in 2017–2019 (*P*<0.001). These testing rates were unchanged across the years of the study. All groups experienced a decrease in LOS in 2020, but this was only found to be significant for patients who speak a language other than English or Spanish (*P*<0.001). None of the patient groups experienced a significant increase in unscheduled returns in 2020 compared to previous years; however, patients who prefer English had significantly more unscheduled returns (7%, 95% CI 6–8%) than patients who preferred Spanish (4%, 95% CI 3–6%) or other languages (2%, 95% CI 0–4%) in 2020 and over the course of the study (Table 5).

## DISCUSSION

This study demonstrates that after adjusting for sex, age, race and ethnicity, there is a strong association between PLOE and COVID-19 diagnoses, where patients with a PLOE had nearly three times the odds of a COVID-19 diagnosis than their English-speaking counterparts. While the overall ED census was lower than normal, the proportion of patients with a PLOE increased, consistent with this association. Use of interpreters, as reflected by the ratio of interpreter contacts per patient with a PLOE, went up during COVID-19, driven largely by the use of video and telephonic interpreters.

Our findings are consistent with other studies that demonstrate the significant impact of COVID-19 on minority communities and PLOE patients,^12^,^18^ and reinforces the specific impact on patients who have a PLOE in the ED population. Various existing studies attempt to account for this strong association. A number of social and systemic factors are likely contributors.^12^ In addition to language differences, members of immigrant communities are more likely to live in larger households and work in industries that do not accommodate remote work.^19^ It has been shown that patients with a PLOE and people who live in large households are more likely to report difficulty in obtaining supplies to safely quarantine.^20^

At a community level, larger household size and percentage of foreign-born persons or non-citizens have been predictive of increased case rates and deaths.^21^ Of note, increased use of public transit has also been found to predict increased death rates in communities.^21^ In addition, patients and families with a PLOE have poorer understanding of public health directives on mask-wearing and shelter-in-place orders and, in contrast to our findings, have significant difficulty accessing interpreters once they have come into contact with the healthcare system.^22^ Given all these factors, the strong association of COVID-19 with language is not surprising.

While the lay press described significant difficulties with accessing interpreters, our overall interpreter usage on a per-patient basis did increase vs previous years, consistent with the findings of Hartford and colleagues.^2^–^6^,^13^ While the use of in-person interpreters decreased substantially, the use of telephone and video interpreters increased, as the hospital made an effort to minimize interpreters’ exposure and decrease use of additional PPE. We hypothesize that the increase in overall interpreter usage is related to the decrease in ED census, allowing clinicians more time to use interpreters appropriately, although further study is warranted. While the overall increased use of interpreters is an encouraging finding, the increased reliance on video and telephonic interpreters and shift away from in-person interpreters, and how this impacted both patients and clinicians, merits further study.

While we did demonstrate that there is a strong association between COVID-19 and patients with a PLOE, other typical measures associated with language such as number of studies performed, LOS, and unplanned return visits yielded more variable results. Testing rates were unchanged over the years of the study, with no significant difference in the number of radiology studies ordered across groups. Patients who preferred Spanish had significantly higher laboratory testing rates in 2017–2019 than patients who preferred English or other languages; however, a median of 4 vs 3 is of unclear clinical significance. All groups experienced a decrease in LOS in 2020; however, this was only found to be significant for patients who spoke a language other than English or Spanish. While unscheduled returns to the ED in 48 hours did not significantly change for each group in April 2020, we were surprised to find that contrary to other studies, unscheduled returns to the ED were higher in English-speaking patients than patients with a PLOE in our ED across all years of the study. We hypothesize that various social, cultural, and access-to-care factors were driving this. Further investigation into the factors contributing to these divergences from national trends over a larger time frame is warranted.

Patients with a PLOE face a host of difficulties in their interactions with the healthcare system, including increased LOS, increased readmission rates, less-robust informed consent, and increased adverse medical events.^24^ Interestingly, similar to unscheduled returns, our baseline relative LOS data did not reflect the discrepancies typical for patients with a PLOE. These were extremely provocative findings; further investigation into these baseline numbers over multiple months and years will be needed to fully understand these relationships and what elements contribute to them.

Considering the data presented here, it is clear that the COVID-19 pandemic disproportionately affected patients with a PLOE in our ED. The overall impact on this community on a broader scale will have significant ramifications beyond what was measured here and should be addressed. To improve or even maintain the care of patients with a PLOE following the COVID-19 pandemic and during future health crises, it behooves emergency clinicians and hospital systems to provide language-appropriate services to patients, and researchers to prioritize inclusion of these patients in clinical trials. In some cases, patients with a PLOE were specifically excluded from some COVID-19 clinical trials.^23^ Moving forward, special attention to language inclusivity, as has been established for sex, race and ethnicity, is essential in clinical trials and research overall.

While our goal was to assess the association of COVID-19 with patients with PLOE during the initial peak of the pandemic, additional longer. term studies are necessary to further understand the impact on this community and the healthcare system at large. Further evaluation of the impact on this community over the course of the entire pandemic, including hospitalization rates and mortality, as well as the effect on clinicians and interpreters can help us to further understand these impacts.

It has been well established that providing care in a patient’s language will improve care via improved communication and comprehension, improved outcomes, and clinical services utilization.^14^,^15^ The inequity that has emerged for patients with a PLOE in the COVID-19 pandemic must be addressed with linguistically and culturally appropriate patient communication, across the spectrum of healthcare. This should include collecting language data from patients and clinicians, providing linguistically and culturally appropriate communication, both at the patient bedside with interpreters or language-concordant care, and in print, media and public health communications from healthcare systems and policymakers.^7^,^25^ Use of the National Culturally and Linguistically Appropriate Standards, both in day-to-day care and in planning for future pandemics and public health crises, is essential.^25^,^26^

## LIMITATIONS

This study has several limitations. First, it is retrospective in nature, and the data was collected for the purpose of patient care rather than this specific analysis. This data is a snapshot in time from a single month during the initial peak of the pandemic; therefore, additional analyses of preceding and subsequent months, over varying levels of COVID-19 prevalence, may yield different results. We chose the month of April as it did represent an early peak in the pandemic, but the choice of this month could have introduced bias. While we did find that the overall proportion of patients with a PLOE presenting to the ED increased, we cannot determine whether it was specifically COVID-19 that drove this increase or rather an increase in this population in the community as a whole. While we did find that patients with a PLOE have nearly three times the odds of a COVID-19 diagnosis than their English-speaking counterparts, we cannot definitively account for all factors that may have contributed to this. We did adjust for sex, age, race and ethnicity, but it is unclear whether this was a reflection of English-speaking patients seeking care preferentially outside the ED.

In addition, patients in our healthcare system self-report their preferred language on their initial presentation, and we cannot be sure of the accuracy of this reporting, given the known difficulties in communication with patients with a PLOE. Neither could we specifically trace when patients inappropriately identified as English-speaking and the clinician did not access language services of any kind.

Additionally, our data on interpreter usage came from two different sources: the in-house, in-person interpreter log; and that of the commercial vendor providing telephone and video language services. There may be discrepancies in their reporting practices that we cannot account for. While we were able to evaluate the total number of encounters with interpreter services, at the time of this study, interpreter encounters were not directly linked to individual patient encounters by our language services department. Therefore, we cannot trace these back to each individual patient, and we cannot assess whether some patients had zero encounters with interpreter services while others had multiple encounters and how this may have affected their care. We anticipate future research studies in which we will be able to link interpreter encounters to specific patient encounters to provide a more robust analysis.

Finally, the COVID-19 pandemic has had a multitude of impacts on our healthcare system, and it would be impossible to account for all of these in our analysis. Patients with a PLOE have many other characteristics that may have impacted their care, including socioeconomic status, education, the amount of time they have lived in the United States, and previous interactions with our healthcare system, to name a few. Because our data was evaluated retrospectively, we cannot analyze for these types of confounders or modifiers.

## CONCLUSION

Our data confirms that COVID-19 disproportionately affected patients who had a preferred language other than English, with patients with a PLOE 2.9 times more likely to test positive for COVID-19 than their English-speaking counterparts. Efforts should be made to mitigate this effect via language-concordant care, professional interpreters. and culturally appropriate interaction and information dissemination, not only as it relates to planning for public health crises, but in the day-to-day function of the healthcare system at large. Continued research into the factors driving these inequities and ways to mitigate them is warranted.

## Supplementary Information





## Figures and Tables

**Table 1:** Demographics of adult emergency department visits in April of each year, 2017–2020.

	2017 N (%)	2018 N (%)	2019 N (%)	2020 N (%)
Total number of visits	4,884	4,812	4,950	3,555
Median Age, years (IQR)	44 (31 – 57)	45 (32 – 58)	45 (31–58)	45 (33–58)
Gender				
Female	2,101 (43)	2,019 (42)	2,087 (42)	1,329 (37)
Male	2,783 (57)	2,793 (58)	2,863 (58)	2,226 (63)
Ethnicity				
Hispanic/Latinx	1,788 (37)	1,707 (35)	1,850 (37)	1,402 (39)
Not Hispanic/Latinx	3,043 (62)	3,046 (63)	3,061 (62)	2,098 (59)
Unknown / Missing	53 (1)	59 (1)	39 (1)	55 (2)
Race				
Asian	64 (1)	84 (2)	85 (2)	63 (2)
Black	784 (16)	695 (14)	725 (15)	492 (14)
Native American	78 (2)	94 (2)	81 (2)	91 (3)
Other Pacific Islander	3 (0)	17 (0)	8 (0)	12 (0)
White	3,613 (74)	3,489 (73)	3,601 (73)	2,474 (70)
Other	298 (6)	387 (8)	411 (8)	373 (10)
Unknown / Missing	44 (1)	46 (1)	39 (1)	50 (1)
Primary Preferred Language				
English	4,156 (85)	4,073	4,142 (84)	2,875 (81)
Spanish	589 (12)	607 (13)	675 (14)	559 (16)
Other	139 (3)	132 (3)	133 (3)	121 (3)
Interpreter Services Encounters	1,142	1,538	1,457	1,346

*IQR*, intraquartile range.

**Table 2:** Patient characteristics of emergency department patient visits by COVID-19 diagnosis in April 2020.

	COVID-19 Diagnosis			

	Yes (n = 261)		No (n = 3,296)	
	
	n	(%)	n	(%)
Median Age, years (IQR)	51	(39 – 61)	44	(33 – 57)
Gender				
Female	164	(63)	1,232	(37)
Male	97	(37)	2,064	(63)
Ethnicity				
Hispanic/Latinx	149	(57)	1,254	(38)
Not Hispanic/Latinx	109	(1)	1,987	(60)
Unknown / Missing	3	(42)	55	(2)
Race				
Asian	8	(2)	54	(2)
Black	30	(11)	464	(14)
Native American	3	(1)	88	(3)
Other Pacific Islander	1	(0)	12	(0)
White	166	(64)	2,297	(70)
Other	50	(20)	330	(10)
Unknown / Missing	3	(1)	51	(2)
Primary Preferred Language				
English	147	(56)	2,727	(83)
Spanish	102	(39)	459	(14)
Other	12	(5)	3	(0)
Area Deprivation Index				
Quintile 1 (least deprived)	17	(7)	359	(11)
Quintile 2	33	(13)	540	(16)
Quintile 3	35	(13)	403	(12)
Quintile 4	80	(31)	829	(25)
Quintile 5 (most deprived)	84	(32)	982	(30)
Missing	12	(5)	183	(6)

IQR, interquartile range.

**Table 3:** Multivariable logistic regression model to evaluate the association between limited English proficiency and COVID-19 diagnosis among emergency department patients.

Variable	OR	(95% CI)
Limited English proficiency* (unadjusted)	3.7	(2.9 – 4.9)
Limited English proficiency (adjusted)	2.9	(2.1 – 4.1)
Age	1.0	(1.0 – 1.0)
Female	0.9	(0.7 – 1.2)
Hispanic	1.4	(1.0 – 2.1)
Race		
Black	1.3	(0.8 – 2.0)
Asian	1.7	(0.8 – 3.8)
Other†	1.3	(0.9 – 1.8)
Area Deprivation Index		
Quintile 2	1.2	(0.6 – 2.2)
Quintile 3	1.3	(0.7 – 2.4)
Quintile 4	1.2	(0.7 – 2.2)
Quintile 5 (most deprived)	1.7	(1.0 – 2.9)

References: English primary preferred language; ethnicity: non-Hispanic; race: White; Area Deprivation Index: Quintile 1 (least deprived).

* Defined as preferring any other language besides English, as reported by the patient.

† Defined as Native American, Alaska Native, Native Hawaiian or other Pacific Islander, or other patient-reported race.

*OR*, odds ratio; *CI*, confidence interval.

**Table 4:** Prevalence ratio of interpretation encounters to ED visits during the month of April 2017–2020.

	Language-line encounters		In-person encounters		Total interpretation encounters	
		
	Prevalence ratio	(95% CI)	Prevalence ratio	(95% CI)	Prevalence ratio	(95% CI)
2017	Reference		Reference		Reference	
2018	1.44	(1.27–1.64)	1.33	(1.22–1.44)	1.37	(1.28–1.46)
2019	1.93	(1.71–2.17)	0.94	(0.85–1.03)	1.26	(1.18–1.35)
2020	3.97	(3.56–4.43)	0.49	(0.43–0.56)	1.62	(1.52–1.73)

*CI*, confidence interval.

**Table 5:** Secondary outcomes.

	2017		2018		2019		2020	
			
	Median	(IQR)	Median	(IQR)	Median	(IQR)	Median	(IQR)
Median number of laboratory studies								
English	3	(0 – 5)	3	(0 – 5)	3	(0 – 5)	3	(0 – 6)
Spanish	4	(1 – 5)	4	(2 – 5)	4	(1 – 5)	4	(1 – 5)
Other	3	(0 – 6)	3	(0 – 5)	3	(0 – 5)	3	(0 – 6)
Median number of radiology studies								
English	1	(0 – 1)	1	(0 – 1)	1	(0 – 1)	1	(0 – 2)
Spanish	1	(0 – 1)	1	(0 – 2)	1	(0 – 2)	1	(0 – 2)
Other	1	(0 – 1)	1	(0 – 2)	1	(0 – 1)	1	(0 – 2)
Median ED length of stay (minutes)								
English	296	(188 – 453)	296	(190 – 453)	272	(171 – 413)	258	(160 – 410)
Spanish	279	(191 – 407)	281	(186 – 388)	265	(179 – 403)	235	(159 – 331)
Other	285	(172 – 414)	249	(155 – 361)	263	(140 – 360)	215	(88 – 307)
Patients with return visit within 48 hours	n	(%, 95% CI)	n	(%, 95% CI)	n	(%, 95% CI)	n	(%, 95% CI)
English	281	(7, 6 – 7)	210	(5, 4 – 6)	268	(6, 6 – 7)	193	(7, 6 – 8)
Spanish	13	(2, 1 – 3)	16	(3, 1 – 4)	25	(4, 2 – 5)	24	(4, 3 – 6)
Other	1	(1, 0 – 2)	3	(2, 0 – 5)	3	(2, 0 – 5)	2	(2, 0 – 4)

*IQR*, interquartile ratio; *ED*, emergency department.

**Table 6:** P-values for the comparison of median number of studies and LOS between English vs. PLOE patients in each year. (Companion to Table 5)

	p-value*	p-value*
Median number of laboratory studies	Spanish	Other
2017	<0.001	0.22
2018	<0.001	0.43
2019	<0.001	0.47
2020	0.08	0.77
Median number of radiology studies	Spanish	Other
2017	0.2822	0.28
2018	0.01	0.92
2019	0.01	0.42
2020	0.12	0.80
Median ED length of stay (minutes)	Spanish	Other
2017	0.02	0.20
2018	0.004	0.0004
2019	0.63	0.03
2020	0.001	<0.001

* Reference=English language

*LOS*, length of stay; *PLOE*, preferred language other than English; *ED*, emergency department.
