# Supplementary material for: Impact of COVID-19 on Patients with a Preferred Language Other than English in the Emergency Department
Source: West J Emerg Med. 2025 Jul 9;26(4):960–9. doi: 10.5811/westjem.18610 (PMC12342450; doi:10.5811/westjem.18610)
Supplement: Supplementary file 1 [file wjem-26-960-g001.pdf]

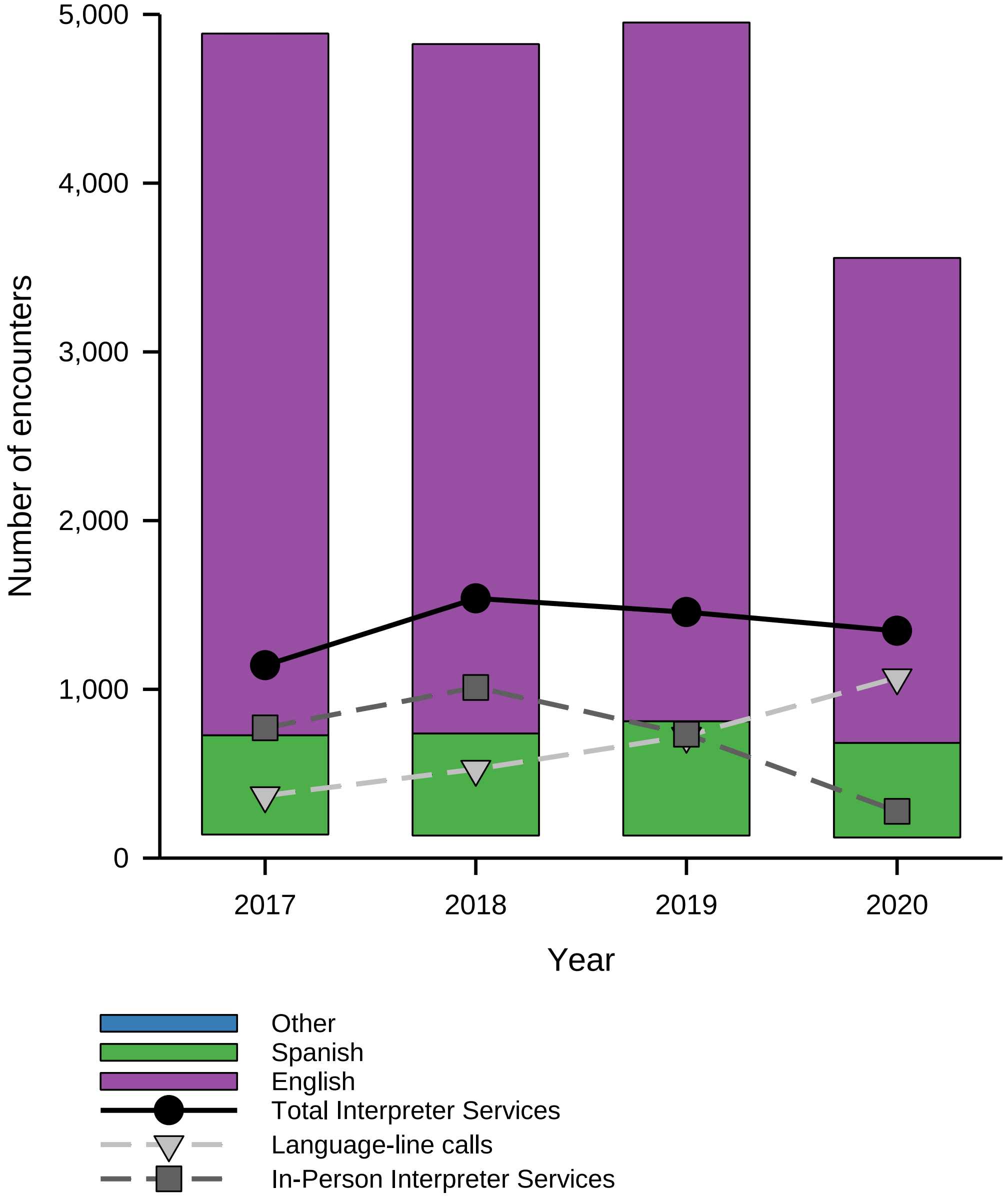

5,000
4,000
3,000
2,000
1,000
0
Number of encounters
2017
2018
2019
2020
Year
Other
Spanish
English
Total Interpreter Services
Language-line calls
In-Person Interpreter Services
