# Supplementary figures and images for: Impact of COVID-19 on Patients with a Preferred Language Other than English in the Emergency Department
Source: West J Emerg Med. 2025 Jul 9;26(4):960–9. doi: 10.5811/westjem.18610 (PMC12342450; doi:10.5811/westjem.18610)

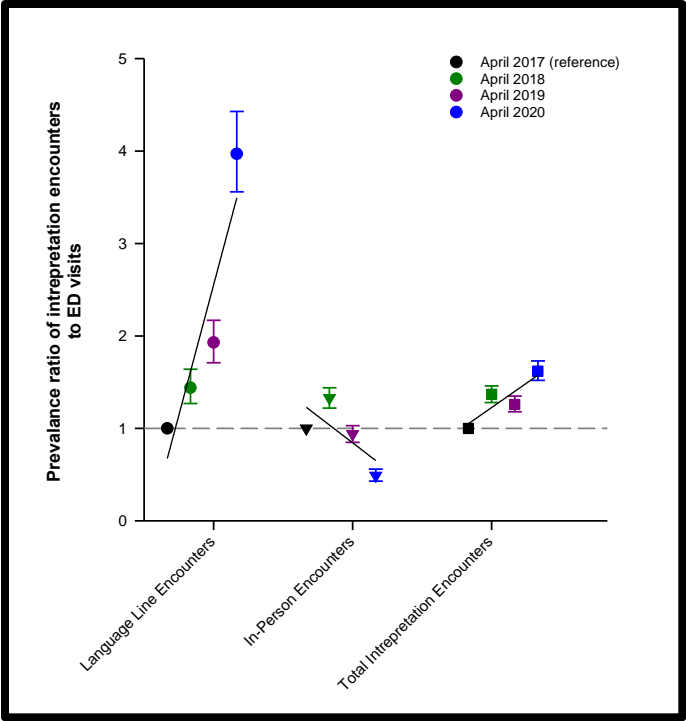

Supplement: Supplementary file 2 [file wjem-26-960-g002.pdf]
